# Impact of a Recombinant Biocontrol Bacterium, *Pseudomonas fluorescens* pc78, on Microbial Community in Tomato Rhizosphere

**DOI:** 10.5423/PPJ.OA.08.2015.0172

**Published:** 2016-04-01

**Authors:** Hyun Gi Kong, Nam Hee Kim, Seung Yeup Lee, Seon-Woo Lee

**Affiliations:** Department of Applied Bioscience, Dong-A University, Busan 604-714, Korea

**Keywords:** gene transfer, microbial community, *Pseudomonas fluorescens*, T-RFLP, tomato rhizosphere

## Abstract

*Pseudomonas fluorescens* pc78 is an effective biocontrol agent for soil-borne fungal diseases. We previously constructed a P43-*gfp* tagged biocontrol bacteria *P. fluorescens* pc78-48 to investigate bacterial traits in natural ecosystem and the environmental risk of genetically modified biocontrol bacteria in tomato rhizosphere. Fluctuation of culturable bacteria profile, microbial community structure, and potential horizontal gene transfer was investigated over time after the bacteria treatment to the tomato rhizosphere. Tagged gene transfer to other organisms such as tomato plants and bacteria cultured on various media was examined by polymerase chain reaction, using gene specific primers. Transfer of chromosomally integrated P43-*gfp* from pc78 to other organisms was not apparent. Population and colony types of culturable bacteria were not significantly affected by the introduction of *P. fluorescens* pc78 or pc78-48 into tomato rhizosphere. Additionally, terminal restriction fragment length polymorphism profiles were investigated to estimate the influence on the microbial community structure in tomato rhizosphere between non-treated and pc78-48-treated samples. Interestingly, rhizosphere soil treated with strain pc78-48 exhibited a significantly different bacterial community structure compared to that of non-treated rhizosphere soil. Our results suggest that biocontrol bacteria treatment influences microbial community in tomato rhizosphere, while the chromosomally modified biocontrol bacteria may not pose any specific environmental risk in terms of gene transfer.

Biological control of plant diseases using various beneficial microorganisms can potentially reduce the disease instances and has been extensively investigated from both, basic and applied points of view (Cheng et al., 2010; Schisler et al., 2004; Wang et al., 2010). Among these beneficial microorganisms, *Pseudomonas* spp. have received much attention as effective biocontrol agents against a broad range of soil-borne diseases (Kim et al., 2004b; Weller, 2007; Whipps et al., 2001). Biocontrol mechanisms employed by *Pseudomonas* spp. include production of antimicrobial compounds, plant growth promotion, production of siderophores, and competition with disease-causing microbes for niches and nutrients (Haas and Keel, 2003; Haas and Défago, 2005; Morales et al., 2010; Özyilmaz and Benlioglu, 2013; Weller, 2007). Previous attempts to search for novel rhizobacteria with biocontrol activity frequently resulted in the selection of *Pseudomonas* spp. as effective organisms for the suppression of plant fungal diseases (Sang et al., 2013).

*P. fluorescens* pc78 strain was initially isolated as a contaminant in fungal cultures, with strong antifungal activity against several plant-pathogenic fungi, especially against rice sheath blight caused by *Corticium sasaki*, suggesting that it could be used as a biocontrol agent (Choi et al., 2006). Because *Pseudomonas* spp. are vulnerable to genetic modifications, genetic engineering has been widely employed to improve their biocontrol activity (Cook, 1993). Some genetically modified recombinant bacteria were observed to have outstanding survivability and enhanced adaptability to wild environmental conditions (Awong et al., 1990). However, the major concern in using genetically modified microorganisms is the potential for the introduced genes to rapidly spread to other organisms in the ecosystem. Although, genetically modified biocontrol agents may have more benefits than the chemical control agents, they pose a number of concerns about the safety of the soil microbial community (Cook et al., 1996). In order to assess the risk posed by genetically modified bacteria in soil environment, the bacterial strains with selectable marker can be utilized. We previously introduced a marker system to monitor bacterial traits *in situ* (Kong et al., 2009). The biomarker was developed by the fusion of P43 promoter of *Bacillus subtilis* (Wang and Doi, 1984), a constitutive promoter, and a promoter-less green fluorescent protein (*gfp*) gene (Miller and Lindow, 1997) by overlap extension polymerase chain reaction (Higuchi et al., 1988, Zhang et al., 2005) followed by cloning in a mini-Tn5 derivative, pTn*Mod*-OKm plasposon (Dennis and Zylstra, 1998). The fusion construct, P43-*gfp*, was successfully integrated into the transposon and the constitutive expression of *gfp* gene under the P43 promoter was tested in *Escherichia coli* DH5a, *B. licheniformis* N1 (Lee et al., 2006), *B. subtilis* 168, and *P. fluorescens* pc78 (Kong et al., 2009). In particular, *B. licheniformis* N1 with P43-*gfp* fusion construct was successfully used to evaluate the bacterial distribution on strawberry plants (Kong et al., 2010).

In the present study, we used *P. fluorescens* pc78 wild type strain and strain pc78-48, carrying P43-*gfp* in its chromosome, to estimate the potential gene transfer among microorganisms and to evaluate the effect of biocontrol strain on the microbial community in tomato rhizosphere. Several strategies are available for microbial community analysis such as temperature gradient gel electrophoresis (TGGE), denaturing gradient gel electrophoresis (DGGE), and massive sequencing of phylogenetic marker genes (Bakker et al., 2002; Mühling et al., 2008; Sokol et al., 2006). In this study, we employed terminal restriction fragment length polymorphism (T-RFLP) which is similar to TGGE and DGGE in terms of amplifying 16S ribosomal RNA gene and subsequent restriction analysis. However, T-RFLP is easier to use for quantitative and qualitative analysis of soil microcosms and has better resolution and reproducibility (Tiedje et al., 1999). High-throughput sequencing of partial 16S rRNA genes by next generation sequencing (NGS) technology followed by community analysis are currently popular for microbial community analysis (Metzker, 2010). However, the NGS based analysis, being costly, is not amenable to many laboratories. Therefore, in the present study, we deployed T-RFLP to evaluate microbial communities.

## Materials and Methods

### Bacterial strains and culture conditions

*E. coli* strains were grown at 37°C on Luria-Bertani (LB) agar or in LB broth supplemented with the appropriate antibiotics. The *P. fluorescens* pc78 and its mutants (Choi et al., 2006; Kong et al., 2009) were routinely grown at 28°C on mannitol-glutamate medium (MG) (Keane et al., 1970) supplemented with 0.25 g/l yeast extract (MGY) or in MG/MGY broth. The plasmid pTnEZG1-1 carrying P43-*gfp* fusion construct (Kong et al., 2009) was maintained in *E. coli* and used to tag *P. fluorescens* p78. Kanamycin (50 μg/ml) was used for selection of *E. coli* and *P. fluorescens* pc78 mutant strains. Microbiological media R2A (Reasoner et al., 1979), trypticase soy agar (TSA), and AIA (Duerden, 1997) were used to grow bacterial population from tomato rhizosphere soils.

### Selection of tagged *Pseudomonas* strain

**Fig. 1:**
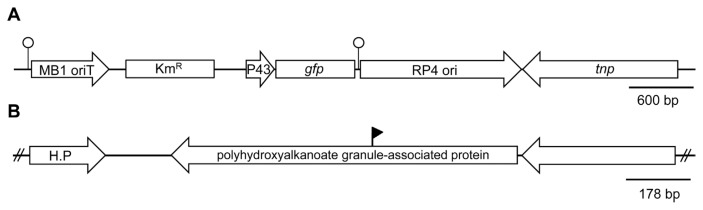
Map of pTnEZG1-1 (A) and plasposon insertion region in the *P. fluorescens* pc78 genome (B). The pTnEZG1-1 was previously constructed by Kong et al. (2009) by integration of P43 promoter fused to a promoterless *gfp* gene into pTnMod-Okm (Dennis and Zylstra, 1998). The open circles represent the invert repeat site of transposon. The solid flag in panel B indicates the insertion site of the pTnEZG1-1. H.P indicates hypothetical protein.

Transposon insertion in *P. fluorescens* pc78 strain was previously conducted using pTnEZG1-1 (Fig. 1A) (Kong et al., 2009). The transposon pTnEZG1-1 was modified from pTn*Mod*-OKm (Dennis and Zylstra, 1998) to carry P43-*gfp*. Among several *gfp-*tagged strains containing P43-*gfp*, pc78-48 was selected, because it exhibited an antifungal activity similar to its wild type, following a dual culture with *Rhizoctonia solani* PY-1 (Heo et al., 2008). For all the other tested traits, pc78-48 displayed phenotypes similar to its wild type; for example, the cell motility of pc78-48, such as swarming and swimming (Rashid and Kornberg, 2000), was also equivalent to that of its wild type strain.

### Recombinant DNA techniques

Recombinant DNA techniques such as plasmid isolation, restriction digestion, DNA self-ligation, plasmid transformation etc. were carried out as described by Sambrook et al. (1989). Genomic DNA of *P. fluorescens* pc78 wild-type and *gfp*-tagged strains were purified using bacterial genomic DNA extraction Kit (Promega, USA). To identify the transposon-inserted site in the *P. fluorescens* strain pc78-48, chromosomal DNA of the strain pc78-48 was completely digested, self-ligated, and subsequently transformed into *E. coli* DH5α, to rescue the plasmid carrying the transposon-inserted DNA. Nucleotide sequence of the rescued recombinant DNA was determined using the primers, Gfp5 and MB ori (Table 1).

### Bacterial applications to tomato rhizosphere

Tomato plants were grown in a plastic house, as per routine cultivation practices, in 15-cm-diameter pots filled with the commercial horticulture nursery media soil (Punong Co. Ltd, Korea). *P. fluorescens* pc78 wild type and strain pc78-48 were pre-cultured for 24 h in casamino acid-peptone-glucose (CPG) broth, the bacterial cell pellet was harvested by centrifugation at 7,000 rpm, and washed with 0.75% NaCl. Final bacterial cell suspension was adjusted to A_600_ = 0.3, using a spectrophotometer (Beckman Coulter, Inc., USA), to have approximately 1 × 10^8^ cfu/ml. The bacterial cell suspension was applied to 3-weeks-old tomato seedlings, at third true-leaf stage. The applied volume of bacterial cell suspension was about 1/10 the volume of soil in the pot. The tomato plants were maintained in the plastic house until the assessment for rhizosphere microbial population and the diversity of rhizosphere microbes.

### Investigation of bacterial gene transfer in tomato plants

In order to investigate if the gene tagged in the bacterial chromosome could be transferred to plants, tomato plants were harvested one month after pc78-48 application. The harvested tomato plants were thoroughly washed with tap water, any surface attached microbes were removed using an ultrasonic cleaner (Branson 5510, Branson, USA), the surface was sterilized with 10% bleach for 15 min, and then extensively washed with sterile water. Total plant DNA was extracted using the manual DNA extraction protocol. Briefly, DNA was isolated from 1 g of finely-ground tomato plants with 500 μl of DNA extraction buffer (100 mM Tris HCl pH 8.0, 500 mM NaCl, 50 mM EDTA) containing 0.7% β-mercaptoethanol and 33 μl 20% SDS. The lysate was vigorously mixed and incubated at 65°C for 15 min and proteins and cell debris were removed by 5 M potassium acetate and phenol: chloroform: isoamylalcohol (25:24:1) extraction. Finally, the DNA was purified by alcohol precipitation and used for PCR investigation to detect P43-*gfp*.

### Investigation of gene transfer to bacterial population in tomato rhizosphere

To examine changes in bacterial population in the tomato rhizosphere treated with *P. fluorescens* strains, the rhizosphere soil was obtained by detaching the soil tightly associated to tomato roots by mild sonication using an ultrasonic cleaner. Soils from three individual tomato plants were pooled on 6, 12, and 30 days after the bacterial application to tomato rhizosphere. Total number of the culturable bacteria was counted by plating serially diluted soil suspension onto the several specific media such as tryptic soy agar (TSA), Reasoner’s 2A agar (R2A), actinomycete isolation agar (AIA), and MG. Bacterial identification up to the level of phylum was conducted by amplifying and sequencing 16S rRNA gene using 27F and 1492R primers (Table 1). Potential gene transfer of P43-*gfp* from strain pc78-48 to rhizosphere microorganisms was examined by PCR and GFP expression. PCR amplification of the culturable bacteria was performed by using P43-2 and Gfp-4 primers (Table 1). pTnEZG1-1 was used as the positive control and kanamycin resistant fluorescent pc78-48 were excluded from the PCR analysis.

Following conditions were used for PCR: an initial DNA template denaturation step at 95°C for 3 min; 30 cycles consisting of denaturation at 95°C for 30 sec, annealing at 50°C for 30 sec, and extension at 72°C for 1 min; and a final extension step at 72°C for 5 min. Meanwhile, GFP expression of the bacterial colonies formed on different media such as TSA, R2A, AIA, and MG were examined by LSM510 confocal laser scanning microscope (Carl Zeiss, Germany). An Argon laser beam (488 nm) was used to excite GFP. Fluorescence signal from GFP were detected using the filters for fluorescein isothiocyanate (FITC; BP 505–530 green).

### PCR and terminal restriction fragment polymorphism (T-RFLP) analysis

To estimate the effect of biocontrol bacteria on the rhizosphere soil microbial community, we conducted T-RFLP analysis of tomato rhizosphere soils treated with *P. fluorescens* pc78 and/or pc78-48. Rhizosphere soils obtained from three individual tomato plants were pooled before metagenomic DNA isolation. Metagenomic DNA from tomato rhizosphere soil was isolated, as described previously (Zhou et al., 1996). Total 16S rRNA gene was amplified using the general bacterial primers 27F (5′-AGAGTTTGATCMTGGCTCAG-3′), with its 5′ end labeled with fluorescein amidite, and 1492R (5′-ACGGYTACCTTGTTACGACTT-3′) for T-RFLP (Table 1). PCR for 16S rRNA gene amplification was conducted at 94°C for 5 min; 30 cycles consisting of denaturation at 94°C for 45 sec, annealing at 57°C for 45 sec, and extension at 72°C for 1 min; and a final extension step at 72°C for 10 min.

The PCR products were digested with the restriction enzymes *Msp*I or *Rsa*I and the terminal restriction fragments (TRFs) were analyzed by DNA sequencing, commercially performed by the DNA sequencing facility of SolGent Corp. (Daejeon, Korea). Digested, fluorescent-labeled fragments were separated on an ABI 377 capillary sequencer (Applied Biosystems, Foster City, CA, USA) and imaged using GeneScan software (Applied Biosystems). T-RFLP data was analyzed by measuring the area of peaks with upper threshold at 30 bp and normalized by relative peak abundance over 0.5%.

Microbial diversity such as species richness and evenness of the bacterial community were estimated by calculating Simpson’s, Shannon, and equitability indices, using the percentage of TRFs area. Normalized T-RFLP data for each sample were transferred into data matrix for statistical analysis. Principal coordinate analysis (PCoA) plot and hierarchical clustering plot were generated using the Bray-Curtis dissimilarity measures. All statistical analyses were performed using the paleontological statistics software (PAST) (Hammer et al., 2001) and R version 3.2.0 (R Core Team, 2013).

## Results

### GFP tagging of Pseudomonas fluorescens pc78

A biocontrol bacterium *P. fluorescens* pc78 was previously tagged using modified transposon pTnEZG1-1 (Fig. 1A) (Kong et al., 2009). A number of transposon-inserted strains of *P. fluorescens* pc78 showed variations in bacterial phenotypes in terms of the following traits: bacterial growth rate, antifungal activity, motility (swimming and swarming), and extracellular polysaccharide production. However, a previously selected pc78-48 strain (Kong et al., 2009) was notably similar to the wild type strain in all the tested bacterial traits. Transposon inserted region of the strain pc78-48 was identified by plasmid rescue and subsequent DNA sequence analysis of the Tn-flanking fragment. DNA sequence analysis revealed that the strain pc78-48 carried transposon in a gene encoding a protein with polyhydroxy-alkanoate granule-associated domain (Fig. 1B).

### Bacterial colonization of pc78-48 in tomato rhizosphere

**Fig. 2:**
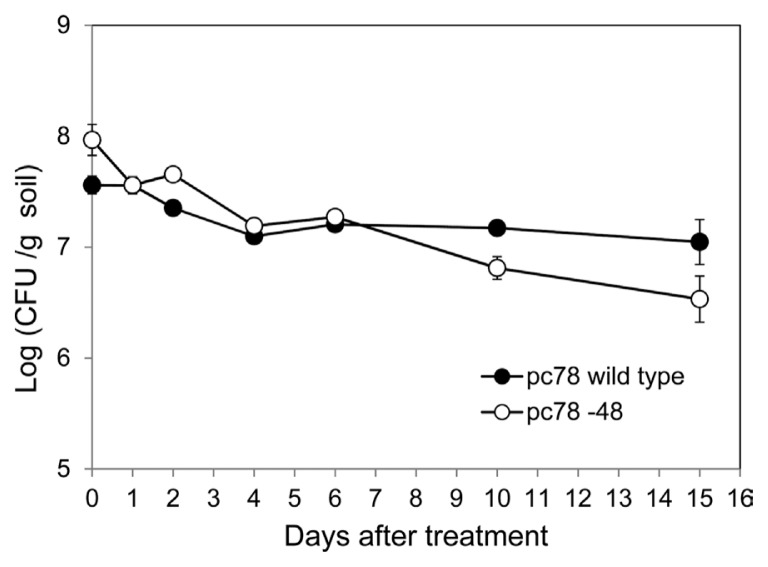
The cell viability was evaluated by dilution plate count of stain pc78 and pc78-48 from 1 g of rhizosphere soils. Vertical bars represent the standard deviation of data from three replicates.

In order to investigate bacterial colonization of *P. fluorescens* strains in tomato plant rhizosphere, bacterial population of pc78 wild type and pc78-48 tagged strain were compared over a period. Both pc78-48 and pc78 strains maintained a population of 10^7^ cfu/g in the rhizosphere soil for 6 days after the treatment. However, bacterial population of strain pc78-48 in the rhizosphere gradually decreased from 15 days onwards after the treatment, whereas that of pc78 strain was stably maintained (Fig. 2). This result suggests that pc78-48 may be less fit to maintain its population in tomato plant rhizosphere than the wild type, although their capability for initial colonization of rhizosphere were not different.

### Bacterial gene transfer to other organisms

**Fig. 3:**
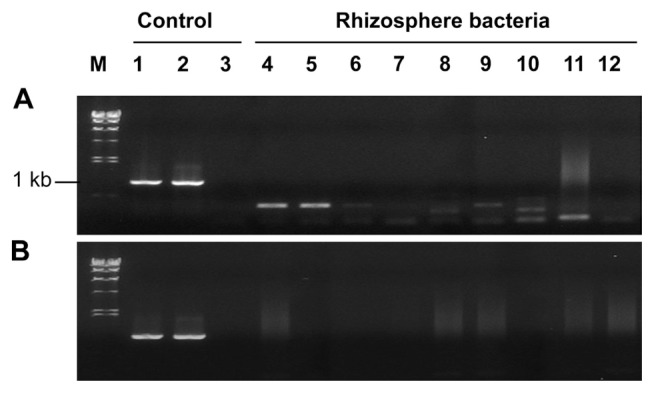
PCR screening of the tagged gene transfer from *Pseudomonas fluorescens* strain pc78-48 to actinobacteria (A), firmicute and proteobacteria (B) which were the selected bacteria from the treated soil. pTnEZG1-1 (Lane 1), *P. fluorescens* pc78-48 (Lane 2), *P. fluorescens* pc78 wild type (Lane 3) used as control.

Horizontal gene transfer is a frequent event among bacterial strains at the inter-species or intra-species level (Darmon and Leach, 2014) and it occurs even in inter-domain level between *Agrobacterium* and plants (Matveeva and Lutova, 2014). Using the tagged strain pc78-48, we investigated gene transfer from pc78-48 to tomato plants or other rhizobacteria by monitoring green fluorescence emission or by PCR amplification of P43-*gfp* gene. Attempts to detect transposon fragment carrying P43-*gfp* by PCR with gfp-5 and MB *ori* gene primers failed to amplify any expected PCR product from the tomato plants (data not shown). Furthermore, we assessed gene transfer from pc78-48 to tomato rhizosphere soil microorganisms. A total of 80 bacterial colonies were randomly selected and briefly identified to the phylum level by 16S rRNA gene sequences. Those bacterial isolates included members of three different phyla, namely, *Proteobacteria*, *Actinobacteria*, and *Firmicutes*, which are dominant in soil. Isolated rhizobacteria were monitored for potential gene transfer from tagged strain pc78-48 to the rhizosphere bacteria. None of isolates exhibited *gfp* expression, and attempts to amplify tagged DNA fragment yielded negative results (Fig. 3) The pTnEZG1-1 and pc78-48 were used as positive control, respectively (lane 1 and lane 2) and pc78 was a negative control on the lane 3 of Fig. 3.

### Fluctuation of microbial community in tomato plant rhizosphere by *P. fluorescens* treatment

**Fig. 4:**
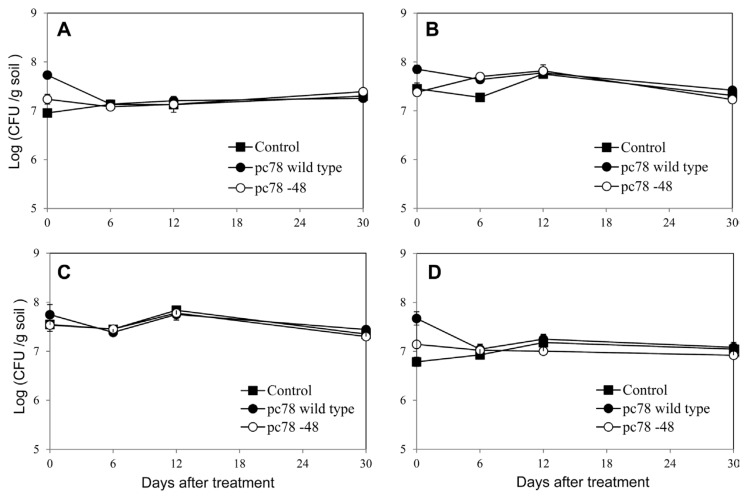
Analysis of the number of cultured bacteria in tomato plant rhizosphere on different media at various times after inoculation of pc78 and pc78-48 stains. AIA medium (A), TSA medium (B), R2A medium (C), MG medium (D). Vertical bars represent the standard deviation of data from three replicates.

Population change of culturable bacteria was evaluated by counting bacterial colony formation using tomato plant rhizosphere soils on various culture media. Bacterial population on the tested media were not significantly changed until 30 days after both pc78 and pc78-48 application (Fig. 4), suggesting that culturable bacterial population of Actinomycetes and oligotrophic and heterotrophic bacteria were not significantly affected by biocontrol bacterial application. Furthermore, culturable bacterial population was not significantly different among non-treated, pc78-treated, and pc78-48-treated rhizosphere soils. Irrespective of the bacterial application, any noticeable changes in bacterial colony types were not recognized (data not shown).

### Bacterial community variation based on T-RFLP profiles

T-RFLP profiles of DNA digested with *Rsa*I or *Msp*I were not significantly different each other, suggesting that either restriction enzymes do not make much difference to analyze the bacterial community structure (data not shown). The TRFs diversity in non-treated and pc78-48-treated tomato plant rhizospheres increased with time for both the samples, when Simpson’s, Shannon’s diversity, and equitability indices were used (Table 2). Both species richness and species evenness increased in tomato rhizosphere over time, irrespective of *P. fluorescens* pc7–48 treatment.

**Fig. 5:**
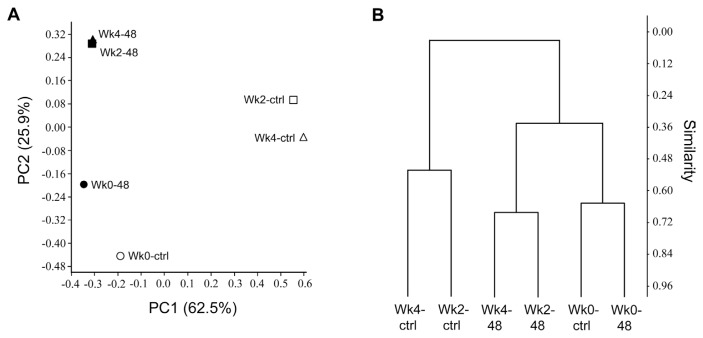
Principal coordinate analysis (PCoA) plots for T-RFLP profiles (A) and a hierarchical clustering plot for all individuals calculated using Bray-Curtis dissimilarity measure (B). * Wk0-ctrl, non-treatment of bacteria in tomato rhizosphere at zero time; Wk2-ctrl, non-treatment of bacteria in tomato rhizosphere at two weeks; Wk4-ctrl, non-treatment of bacteria in tomato rhizosphere at one month; Wk0–48, before pc78-48 application to tomato plant rhizosphere; Wk2–48, two weeks after pc78-48 application; Wk4–48, one month after pc78-48 application.

Principal coordinate analysis (PCoA) and hierarchical clustering plots of the samples revealed that the bacterial community profiles of pc78-48-treated samples (Wk2–48 and Wk4–48) were different from those of the non-treated samples (Wk2-ctrl and Wk4-ctrl), over time (Fig. 5A). The variations in microbial community and community structure was explained by principle component (PC) 1 and PC2 with 62.5% and 25.9% variation, respectively. Rhizosphere microbial community in pc78-48 treated and non-treated samples was separately clustered, indicating the differences in the community structure. The T-RFLP profile was also analyzed by cluster analysis using Bray-Curtis dissimilarity measure. The microbial community (Wk2-ctrl and Wk4-ctrl, Wk2–48 and Wk4–48) from tomato rhizosphere treated for two weeks and one month with pc78-48 and non-treated rhizosphere, were clustered separately from each other, according to their similarities (Fig. 5B). The analysis revealed the same results as seen with ß-diversity analysis by PCoA. Microbial community at zero time, in both the soils (Wk0-ctrl and Wk0–48), were similar to each other according to Bray-Curtis dissimilarity index, suggesting the shift in microbial community by *P. fluorescens* pc78-48 treatment.

## Discussion

Development of molecular biological techniques makes it possible to construct the gene probing tools for analysis of bacterial behavior in soil microbial community (Liu et al., 2010; Oh et al., 2004). In the present study, we developed a monitoring system that can potentially be applied to the study of bacterial behavior in tomato plant rhizosphere. The P43-*gfp* fusion construct, pTnEZG1-1, was introduced in *B. licheniformis* N1 to detect bacteria survival in strawberry plants. Previously, *gfp*-labeled strain N1 successfully exhibited green fluorescence and could be detected by CLSM (Kong et al., 2010). Therefore, we introduced pTnEZG1-1 plasposon into *P. fluorescens* pc78 to generate mutant pc78-48, which showed the same phenotype as wild type strain, such as, antifungal activity and motility. The chromosomal locus of plasposon insertion was identified; the P43-*gfp* insertion site was in the open reading frame encoding polyhydroxyalkanoate (PHA) granule-associated protein domain. This gene is known to encode a protein associated with PHA granule when *Pseudomonas* strains accumulate poly(R)-3-hydroxyalkanoate under limited nutrient conditions (Prieto et al., 1999), though its exact role in bacteria remains unclear.

Bacterial population of the mutant pc78-48, when compared with that of wild type, was slightly reduced in tomato plant rhizosphere at 15 days. PHA is the storage carbon in various bacteria and may help starving bacteria to persist under various environmental conditions. Whether the lack of PHA associated protein in bacteria affects its persistence in the plant rhizosphere is not known, so far. Therefore, we cannot exclude the possibility that the decreased pc78-48 population in the rhizosphere was due to the disruption of PHA granule associated protein. We do not know if *P. fluorescens* pc78 produces PHA or if pc78-48 was affected in PHA production. Since most of phenotypes of pc78-48 were not notably distinguishable from those of wild type pc78 strain, we predict that the disruption of PHA associated protein in pc78 strain would not significantly affect the bacterial capability to initiate rhizosphere colonization. Elaborate gene expression of *Pseudomonas* species in a stationary phase or soil condition would regulate bacterial persistence in the natural ecosystem (Kim et al., 2014a; Kim et al., 2014b).

Our results apparently exhibited that pc78 or pc78-48 treatment did not affect the bacterial diversity and population much in a culture-dependent analysis. This result was in agreement with those of Donegan and Seidler (1999). In addition, a tagged gene transfer was not noticeable from strain pc78-48 to other rhizosphere microorganism, suggesting that the chromosomally integrated genes are rather stably maintained and not subject to frequent mobilization compared to plasmid- or transposon-borne genes.

Although, the pc78 or pc78-48 treatment in tomato rhizosphere did not affect bacterial population or diversity in a culture-dependent analysis, uncultured bacteria have been demonstrated to constitute the majority of soil microbial ecosystem and plant rhizosphere (Amann et al., 1995; Hugenholtz and Pace, 1996). Therefore, we further analyzed the effect of biocontrol organism application on the tomato rhizosphere microbial diversity in a culture-independent analysis using T-RFLP. Microbial community analysis, based on T-RFLP analysis, has been successfully applied in various microbial ecosystem (Scala and Kerkhof, 2000; Osborn et al., 2000). Bacterial community in tomato rhizosphere treated with the biocontrol bacterium pc78-48 was significantly different from that in the non-treated tomato rhizosphere (Fig. 5). This is likely because the culture-dependent analysis of bacterial populations only deals with the minor fraction of total microorganisms in rhizosphere. To better understand the microbial community shift by introducing biocontrol organism in tomato rhizosphere, further analysis of microbial diversity may be necessary. Consequently, this study has successfully investigated the effect of genetically modified biocontrol bacterial treatment in tomato rhizosphere. This study also highlighted the potential gene transfer and microbial community structure.

## Figures and Tables

**Table 1:** Primer pairs used in the study

Primer	Fluorescent label	Sequence (5′ to 3′)	Reference
Gfp-5	None	GCTGGGATTACACATGGCAT	This study
MB ori	None	TTTTGCTCACATGTTCTTTCCTG	This study
P43-2	None	TGATAGGTGGTATGTTTTCGC	This study
Gfp-4	None	ATGCCATGTGTAATCCCAGC	This study
27F	FAM (Blue)	AGAGTTTGATCMTGGCTCAG	This study
1492R	None	ACGGYTACCTTGTTACGACTT	Harrington and On, 1999

a 6-carboxyfluorescein

**Table 2:** Microbial diversity in tomato rhizosphere based on T-RFLP profiles. Species diversity and evenness indices were indicated as Simpson’s index of diversity, Shannon’s index, evenness index, and equitability index

Indices/Treatments	Wk0-ctrl	Wk2-ctrl	Wk4-ctrl	Wk0–48	Wk2–48	Wk4–48
Simpson	0.5472	0.6461	0.8292	0.5844	0.6539	0.7636
Shannon	1.139	1.205	2.237	1.176	1.394	1.686
Evenness	0.3903	0.8338	0.6244	0.463	0.5756	0.6744
Equitability	0.5475	0.8689	0.8261	0.6043	0.7162	0.8106

*Wk0-ctrl, non-treatment of bacteria in tomato rhizosphere at zero time; Wk2-ctrl, non-treatment of bacteria in tomato rhizosphere at two week; Wk4-ctrl, non-treatment of bacteria in tomato rhizosphere at one month; Wk0–48, before pc78-48 application to tomato plant rhizosphere; Wk2–48, two weeks after pc78-48 application; Wk4–48, one month after pc78-48 application.
